# Self-medication with antibiotics in Georgian population

**DOI:** 10.3389/fphar.2024.1254817

**Published:** 2024-02-21

**Authors:** Marina Darakhvelidze, Iagor Kalandadze, Nino Mirzikashvili, David Tsereteli, Natalia Zakareishvili, Ivane Ketchakmadze

**Affiliations:** ^1^ Faculty of Natural Sciences and Medicine, Ilia State University, Tbilisi, Georgia; ^2^ National Centre of decease Control and Public Health, Tbilisi, Georgia; ^3^ United Nations Population Fund, Tbilisi, Georgia

**Keywords:** Georgian population, cross-sectional study, antimicrobial resistance, nonprescription use of antibiotics, self-medication prevalence

## Abstract

**Background:** Self-medication with antibiotics is a global phenomenon and a potential contributor to human pathogen resistance to antibiotics. It involves obtaining medication without a prescription, taking medicines based on the advice of friends and relatives, or previous treatment experience. Self-medication is common in both developed and developing countries; however, the prevalence of self-medication is higher in developing countries. The aim of this study was to determine the characteristics of antimicrobial self-medication in Georgia and its potential to influence the overall situation regarding antimicrobial consumption in the country.

**Methods:** We conducted a cross-sectional study using a random sampling method and developed a self-administered questionnaire to collect the data. The survey was conducted via the Internet using the Google Forms platform.

**Results:** The overall number of respondents was 742 adults living in Georgia. The results showed that 23.8% (*n* = 177) of adults had consumed antibiotics without a doctor’s prescription, and 12.7% (*n* = 94) confirmed the use of antibiotics by their own decision to treat minor family members. The total prevalence of self-medication was 32.6%. The data analysis revealed a correlation between factor F1 (“personal experience”) and gender (*p* = 0.042, F = 2.6), and between age and factor F2 (“lack of trust in medical practitioners”) (*p* = 0.047, F = 2.691). The correlation was stronger among young adults (aged 18–24) and senior adults (aged 60+). The correlation between the level of education and factor F2 was stronger (*p* = 0.00; F1 = 7.9) than with factor F1 (*p* = 0.04; F = 2.2).

**Conclusion:** Self-medication is prevalent in Georgia; pharmacies are the main sources of antimicrobials. No correlation was found between factor F2, pertaining to “lack of trust in medical practitioners” and gender, between age and factor F1, linked to “personal experience.” The study uncovered a lack of knowledge about self-medication with antibiotics and emphasized the importance of public awareness campaigns and implementing effective interventions to regulate the sales of antibiotics without a doctor’s prescription.

## 1 Introduction

Self-medication has been defined as the consumption of medication (modern and/or traditional) for self-treatment without consulting a medical practitioner either for diagnosis, prescription, or medical supervision (Hughes et al., 2001; Zafar et al., 2008). It involves obtaining medication without a prescription, also taking medicines on the advice of friends and/or relatives, or using previous treatment experience (Klemenc-Ketis et al., 2010; Adeel et al., 2023). In practice, it also includes the use of the medication prescribed to other family members, especially when the treatment of children or older persons is involved. To use a non-prescription medicine safely and effectively, some procedures should be performed normally by a physician treating a patient with prescription medicines. These procedures include accurate assessment and identification of symptoms, setting therapeutic objectives, selecting a product to be used, determining medication dosage and dosage regimen, considering the person’s medical history, contraindications, concomitant medical conditions, and concurrent medications, as well as monitoring the response to the treatment and possible adverse effects (World Health Organization, 2000; Adeel et al., 2023). Self-medication with antibiotics is a global phenomenon and a potential contributor to antibiotic resistance in human pathogen (Friedman and Whitney, 2008; Rather et al., 2017). Self-medication is common in both developed and developing countries; however, the prevalence of self-medication is higher in developing countries, due to increased availability of drugs without a prescription (Klemenc-Ketis et al., 2010; Ahmed and Sulaiman, 2016).

According to a report by WHO, the prevalence of self-medication with antibiotics in developing countries is due to several factors, particularly to limited access to healthcare, accessibility of antibiotics sold as over-the-counter drugs, lack of regulatory strategies, and higher rate of prevalence of communicable diseases as compared to developed countries (Ebert, 2007; Vila and Pal, 2010). The reasons for the wide variation in the prevalence of the self-medication practice may also be differences in social determinants of health, tradition, and culture (Nepal and Bhatta, 2018). In developed countries with well-implemented prescription regulations, self-medication with antibiotics is still observed in certain ethnic groups. For example, in a Hispanic neighborhood of New York City, antimicrobial drugs are available without a prescription. In Europe, studies describing self-medication and storage of antimicrobial drugs in Spain, Greece, Russia, and Malta also suggest considerable use of the drugs without consulting a physician. Prevalence rates for actual self-medication were highest in Eastern Europe (in particular, Romania and Lithuania), followed by Southern Europe (Malta, Spain, and Italy) (Väänänen et al., 2006; Grigoryan et al., 2010). Prevalence of resistance is positively correlated with outpatient prescription medication on a national level (Albrich et al., 2004; Goossens et al., 2005). However, actual consumption of medication may also include self-medication, i.e., using medication obtained without a prescription (Thomas et al., 1998). A mandatory electronic prescription system has been introduced in Georgia, and there are enforcement mechanisms in the form of fines, although there is a suspicion that prescription drugs, including antibiotics, are still being sold without a prescription from pharmacies (World Health Organization, 2019). The information on self-medication with antibiotics in developed countries is nowadays limited. Several studies indicate considerable use of leftovers (Ceaser and Wurtz, 2000; Richman et al., 2001; Grigoryan et al., 2006; Grigoryan et al., 2010), medications shared among family members, antibiotics purchased from a pharmacy, or a source outside the country (Larson et al., 2003; Vanden Eng et al., 2003) in the United States and a few European countries. In addition, little information is available on factors that put a person at-risk for self-medication. Unfortunately, Georgia is not an exception. No research data is available about self-medication with antibiotics in Georgia.

## 2 Aim and objectives of the study

The current study aimed to investigate the peculiarities of self-medication with antibiotics in Georgia, and to determine its potential impact on the use of antimicrobials in the country, as well as to explore the factors that lead to self-medication, to facilitate the development of specific interventions.

The research objectives included: 1) determining the prevalence of self-medication with antimicrobials; 2) identifying factors that could influence self-medication practices; 3) examining the relationship between detected factors and socio-demographic factors; 4) identifying the sources of antimicrobials; 5) identifying information sources on antimicrobials used in self-medication; 6) identifying the reasons for self-medication.

## 3 Materials and methods

### 3.1 Study design

A cross-sectional study design was used to conduct the given study.

### 3.2 Study participants and duration

The study was conducted from 1 July 2022 to the end of August 2022 (during 2 months). The subjects of the study were adults living in Georgia who had used an antimicrobial agent for outpatient treatment of himself/herself or a family member (including a minor). The marginal recall period was 1 year.

### 3.3 Sampling technique

We used probabilistic, simple random sampling (systemic). The sampling frame was drawn up in such a way that the adult residents of the regions of Georgia and the capital city were proportionally included in it. This was carried out according to the scheme developed during meetings with colleagues from different regions.The sample size calculation was based on the following formula: sample size *n* = [DEFF*Np(1-p)]/[(d2/Z21-α/2*(N-1)+p*(1-p)] (Z = confidence level of 95% = estimated prevalence 0.5, d = precision or margin of error allowed in this study–a degree of precision 0.05 was used), *n* = 385, however, taking into account the expected results related to the quality of self-administered questionnaires, we processed the responses of all respondents who participated in the survey.

### 3.4 Inclusion criteria

Adults aged 18 and older.

### 3.5 Exclusion criteria

Healthcare workers (doctors, nurses, pharmacists, medical students).

### 3.6 Questionnaire and data collection

We collected data using a self-administered questionnaire specially developed for this purpose. The questions were mostly simple, closed-ended questions. In addition to the demographic part, the questionnaire contained neutral behavioural questions related to the characteristics of the use of antibiotics for oneself and a minor member of the family (antimicrobials used, frequency of use, reasons, source, *etc.*). We also focused on various aspects of antibiotic consumption for outpatient treatment, such as the availability of a family doctor, doctor visits, prescription practices, and the purchase of antibiotics from pharmacies, among others. The population was surveyed through the Google Forms platform. We delivered the questionnaire (link) via the Internet.

### 3.7 Statistical analysis of data

We analysed the data using the Statistical Package for the Social Sciences (SPSS) version 23 and presented data in frequency, percentage, mean, and standard deviation with the aid of charts and tables. Multivariate analysis (MVA) was performed using a one-way ANOVA and factor analysis. The level of significance was set at *p*-value <0.05.

### 3.8 Limitations

We employed a cross-sectional study design, and therefore causal relationships between variables cannot be established. We also used close-ended questions, that offer respondents a limited set of responses to choose from. In addition, the analyses were based on a self-report creating the risk of over/underreporting.

## 4 Results

Adult citizens (*n* = 742) of Georgia participated in the survey (the data is weighted on demographic variables such as gender and age, based on the results of the 2014 General Population Census; see Supplementary Table 1, Appendix 1): 53.4% of study participants were female, 46.6% were male. 18.7% of them were 18–24 years old, 25.5% were 25–40 years old, 32.2% were 41–60 years old, and 23.6% were over 60 years old (see Supplementary Table 2, Appendix 1). Most respondents were ethnic Georgians (98.4%) (see Supplementary Table 3, Appendix 1). 62% of the respondents were married (see Supplementary Table 4, Appendix 1), and 45.2% of them lived with a child under the age of 18 (see Supplementary Table 5, Appendix 1). 78% of the respondents (*n* = 742) were employed. 19% of them had a monthly income of more than GEL (Georgian Lari) 3,000, 30.4% had an income of GEL 1,000–3,000, 32.5% had an income of GEL 500–1,000, and 18.1% had an income of less than GEL 500 (see Supplementary Tables 6, 7, respectively, Appendix 1). 62.5% of the respondents (*n* = 742) had health insurance (see Supplementary Table 1, Appendix 2). Approximately the same number of respondents (38.7%) said that they were the beneficiaries of the State Universal Health Care Programme or other state programmes (see Supplementary Table 2, Appendix 2). It should be noted that 13.2% (*n* = 98) of the respondents did not know whether they were covered by any state programme. 73.1% of the respondents reported using the services of a family doctor (see Supplementary Table 3, Appendix 2).

The survey showed that 12.2% of respondents had taken an antibiotic during a month preceding the date of their participation in the survey (see Supplementary Table 4, Appendix 2); 23.6% of them—during the last 6 months, 12.3%—during the year, 33.3%—more than a year ago, 16.9% could not recall taking any antibiotics, 1.8% of the respondents said that they had never taken any antibiotic. 87.5% of the 742 respondents agreed that antibiotics should be taken as prescribed by a doctor, and only 12.5% disagreed with this opinion (see Supplementary Table 9, Appendix 2).

More detailed analysis of the results of the study showed that, in 23.8% of cases (*n* = 177) the antibiotics were not prescribed by a physician (see Supplementary Table 5, Appendix 2), however, when antibiotics were prescribed by a doctor (76.2%; *n* = 565), only in 39.2% of cases the patients had received instructions and advice regarding taking the antibiotic (see Supplementary Table 8, Appendix 2), and only 48.6% of them (*n* = 361) had been given a written prescription (see Supplementary Table 6, Appendix 2). Among the patients who had received a doctor’s prescriptions (*n* = 565) only 39% (*n* = 289) used the prescriptions when purchasing antibiotics at pharmacies (because the pharmacist did not request them, or the respondents could not specify the reason) (see Supplementary Table 7, Appendix 2).

Majority of the respondents (63.8%) who used antibiotics without a doctor’s prescription (*n* = 177) made the decision themselves. 17.9% of them had received antibiotics on the advice of a family member, a friend, or a neighbour, who had received medical education but were not medical practitioners (pharmacists or nurses), and 18.3% had received antibiotics on the advice of a family member, a friend or a neighbour who had no medical education (see Supplementary Table 10, Appendix 2). In the above-mentioned cases, the respondents either bought an antibiotic in a pharmacy (64.7%), used the leftover medicine prescribed to a family member (10.4%), or were given the medicine by a friend/neighbour (2.6%), in other cases the source is unknown (respondents do not remember) (see Supplementary Table 11, Appendix 2).

The respondents (*n* = 177) used antibiotics without a doctor’s prescription for respiratory system diseases (39.3%), dental injuries/diseases (3.9%), gastrointestinal diseases (2.6%), and genitourinary infections (0.9%). In isolated cases, antibiotics had been used for gynaecological, dermatological, viral, otologic problems, as well as for high temperature, cold and even headache (see Supplementary Table 12, Appendix 2).

Of the 177 respondents, who had used antibiotics without a doctor’s prescription, most of them (80%, 8%) had used oral antibiotics, and only 1%, 7% of these respondents had used parenteral antibiotics. 17%, 5% of respondents could not recall consuming antibiotics during the study period (see Supplementary Table 13, Appendix 2).

When asked about the criteria for choosing an antibiotic, 25.4% of the 177 respondents, addressed medical personnel for advice, 28%, 8% read the instructions for the medication; 32%, 8% had used the medication before, 2%, 8% followed their neighbour’s/friend’s recommendation; 5%, 1% used antibiotics that were prescribed by a doctor. (see Supplementary Table 14, Appendix 2).

Only 54 respondents (30%) correctly identified the medication used during the treatment under different trade names: semi-synthetic penicillin (*n* = 19), macrolides (*n* = 21), sulfonamides (*n* = 2), fluoroquinolones (*n* = 3), semi-synthetic tetracyclines (*n* = 1).

### 4.1 Use of antibiotics for treatment of health conditions in minors

The majority (*n* = 742) of the interviewed persons (84.4%) denied having used an antibiotic for the treatment of a minor family member, 2.9% could not recall such a fact, and 12.7% (*n* = 94) confirmed the fact of having used antibiotics for treating a health condition of a minor family member by their own decision (see Supplementary Table 15, Appendix 2). Of these, 9.1% used antibiotics during the month preceding the date of their participation in the survey; 32.5%—during the last 6 months; 12.4%—during the last year; and 29.6%—more than a year ago (see Supplementary Table 16, Appendix 2). 16.5% could not remember the exact date of using antibiotics for treating health conditions in minors. 91.2% of antibiotics used were oral, and 4.8% were parenteral (see Supplementary Table 17, Appendix 2).

For the treatment of medical conditions in minors, 73% of the interviewees (*n* = 94) used antibiotics for respiratory system diseases, 4.1% for dental diseases/injuries, 5.2% for diseases of the gastrointestinal system, 9.2% for conditions affecting the genitourinary system, and 8.0% did not remember the reason. In a few cases, antibiotics had been used for neurological, otologic, cardiovascular, infectious diseases. There was also a case of an antibiotic being used to treat a headache (see Supplementary Table 18, Appendix 2).

Only 42 respondents (45%) correctly identified the antibiotic used during the treatment under different trade names: semi-synthetic penicillin (*n* = 20), cephalosporins (*n* = 7), macrolides (*n* = 12), sulphonamides (*n* = 1), semi-synthetic tetracyclines (*n* = 1), chloramphenicol (Levomycetin) (*n* = 1).

In total, the fact of using antibiotics by their own decision for patients of all ages was reported by 32.6% (*n* = 242) of the respondents (*n* = 742) (see Supplementary Table 19, Appendix 2). 40.2% of the respondents reported that the results of treatment with antibiotics without a doctor’s prescription had met their expectations (for example, the treatment ended with a recovery), 58.7% of the respondents were unsure about the outcome (“I don’t know”), only 1.1% reported that the results of the treatment did not meet their expectations (see Supplementary Table 20, Appendix 2).

The respondents who use antibiotics without a doctor’s prescription consume antibiotics less frequently: once every few years (53.9%), or once a year (23.6%), rather than more frequently: every month (2.8%), or once every 3–6 months (3.5%) (see Supplementary Table 21, Appendix 2).

More than half of the respondents (51.4%, *n* = 742) believe that antibiotics are used to treat diseases caused by bacteria, 16.3% believe that they are necessary for the treatment of viruses, and 1.3%—for infectious diseases caused by fungi, 0.9%—for parasites, 20.3% believe that all answers are correct, and 9.9% do not know which of the answers was correct (see Supplementary Table 22, Appendix 2).

Most of the respondents (54.8%; *n* = 742) think that, the development of antibiotic resistance is mostly facilitated by the use of antibiotics without a doctor’s prescription, 55.4% also believe that the use of the wrong doses of antibiotics contributes to this problem, 49.9% of the respondents named the use of antibiotics for the wrong duration as the reason, and 63.1% consider the use of an incorrectly selected antibiotic to be the cause of antibiotic resistance; 11.8% of respondents did not know the answer to the question (note: respondents could select more than one answer to this question) (see Supplementary Table 23, Appendix 2).

The final part of the questionnaire was intended for those who had experienced self-medication with antibiotics for the treatment of themselves or a minor living with them (*n* = 242). This part was processed accordingly. However, this part of the questionnaire (possibly due to neglecting the condition in the instructuon to this part) was completed by 551 (±10) respondents. If we consider the stable number of respondents who “strongly disagree” with certain opinions of the statements offered in this part of the questionnaire (35 %± 5%; number of respondents—150 ± 20), they might be among the respondents who declared that they only used antibiotics when prescribed by a doctor.

Only 2.7% of the respondents who self-medicated with antibiotics (*n* = 242) completely agreed and 17.6% of them mostly agreed that they used antibiotics on their own due to the lack of time to visit a doctor; 29.1% of the respondents neither agreed nor disagreed with the statement; 31.9% of the respondents disagreed, and 18.6% of them strongly disagreed with the statement (see Supplementary Table 24, Appendix 2). 2.6% (*n* = 242) (strongly agreed) and 19.9% (mostly agreed) of the respondents associated the self-medication with antibiotics with the fact that they are easily (for example, without prescription) available in pharmacies. 31.4% of the respondents neither agreed nor disagreed with the statement; 31.2% of the respondents “disagreed” and 14.9% of them strongly disagreed with the statement (see Supplementary Table 25, Appendix 2).

About a fifth (19.6%) of the respondents agreed that they used antibiotics on their own due to a high cost of visiting a doctor and 8% (*n* = 242) of them strongly agreed to the statement; 29% of the respondents neither agreed nor disagreed with the statement; 28.1% of the respondents disagreed, and 15.3% of them strongly disagreed with the statement (see Supplementary Table 26, Appendix 2). 4.4% of the respondents with the experience of self-medication with antibiotics (*n* = 242) strongly agreed and 26.9% of them mostly agreed that they used antibiotics for simple symptoms of illness; that is, they do not consider it necessary to consult a doctor for this reason. 25.5% of them neither agreed nor disagreed with the statement; 26.6% of the respondents “disagreed”, and 16.5% of them strongly disagreed with the statement (see Supplementary Table 27, Appendix 2).

More than half of the respondents with the experience of self-medication with antibiotics (*n* = 242) confirmed that they used antibiotics based on their early experience (6% strongly agreed and 46.2% mostly agreed). 19% of them neither agreed nor disagreed with the statement; 20% of the respondents disagreed, and 8.8% of them strongly disagreed with the statement (see Supplementary Table 28, Appendix 2).

Only 2% of the respondents who self-medicated with antibiotics (*n* = 242) strongly agreed and 10.5% of them mostly agreed that they used antibiotics due to the lack of trust in medical practitioners. 22.7% of the respondents neither agreed nor disagreed with the statement; 42.9% of the respondents disagreed, and 21.9% of them strongly disagreed with the statement (see Supplementary Table 29, appendix 2).

The respondents who self-medicated with antibiotics (*n* = 242) strongly agreed (2.9%) and 19.4% mostly agreed that they used antibiotics for diarrheal diseases (to treat themselves or a minor living with them). 27.7% of the respondents neither agreed nor disagreed with the statement; 37% of the respondents disagreed and 12.9% of them strongly disagreed with the statement (see Supplementary Table 30, Appendix 2).

About one-third of the above-mentioned category of the respondents (*n* = 242) indicated that they used antibiotics to treat a sore throat/cold/cough, “to avoid further complications of the disease” (4.5% strongly agreed and 30.3% mostly agreed); 20.5% of the respondents neither agreed nor disagree with the statement; 31.1% of the respondents disagreed and 13.7% of them strongly disagreed with the statement (see Supplementary Table 31, Appendix 2). 2.3% of the respondents (*n* = 242) strongly agreed and 16.3% of them mostly agreed that they used antibiotics in case of genitourinary infections. 23.1% of the respondents neither agreed nor disagreed with the statement. 38% of the respondents disagreed and 20.3% of them strongly disagreed with the statement (see Supplementary Table 32, Appendix 2).

Most respondents who self-medicated with antibiotics (*n* = 242), disagreed (37.2%) or strongly disagreed (34.8%) that antibiotics should be used “for disease prevention”; 16.8% of the respondents neither agreed nor disagreed with the statement. 7.6% of the respondents mostly agreed and 3.7% of them strongly agreed with the statement (see Supplementary Table 33, appendix 2).

The interviewees who agreed with the above-mentioned statement considered the use of antibiotics acceptable for the purpose of a disease prevention in case of oral and dental infections, upper respiratory tract infections, gastrointestinal pathologies, genitourinary tract illnesses, viral diseases, inflammation, cold, cough, sore throat.

We conducted a factor analysis on the variables of part 4 of the questionnaire. As a result, the variables were grouped into 2 factors, the first of which (F1) was conventionally titled as “personal experience” (questions 1 to 5, inclusive, and question 7), and the second (F2) as “lack of trust in medical practitioners” (question 6 and questions 8 to 10, inclusive) (see Supplementary Table 8, Appendix 1).

The above-mentioned factors were analysed against demographic factors such as gender, age, and education. For this purpose we used the one-way ANOVA test. The analysis revealed a correlation between factor F1 and gender (*p* = 0.042, F = 2.6), but not between factor F2 and gender (*p* = 0.314, F = 1.018) (see Supplementary Table 9, Appendix 1). The correlation was stronger between women and F1 (see Supplementary Table 10, Appendix 1).

When studying the correlation with age groups, a correlation was revealed between the age and the factor F2 (*p* = 0.047, F = 2.691). No correlation was revealed between the age and the factor F1 (*p* = 0.984; F = 0.52) (see Supplementary Table 11, Appendix 1).

However, the correlation of the factor F2 was stronger with the 18–24 and >60 age groups and weaker with the 41–60 and 25–40 age groups (see Supplementary Table 12, Appendix 1).

The correlation between the level of education and both factors is important. The study revealed a stronger correlation of the level of education with the factor F2 (*p* = 0.00; F1 = 7.9) than with the factor F1 (*p* = 0.04; F = 2.2) (see Supplementary Table 3, Appendix 1). In addition, the study revealed a relatively stronger correlation between the factor F1 and secondary vocational education and higher education (bachelor’s degree), and between the factor F2 and secondary vocational education and incomplete higher education (see Supplementary Table 14, Appendix 1).

### 4.2 Discussion

Self-medication is a common healthcare practice in Georgia. As an Eastern European country, the probability of self-medication was higher in Georgia than in other European regions (Grigoryan et al., 2006; Grigoryan et al., 2010). This study found that nearly a third of the nation’s adult population self-medicate with antibiotics to manage their health conditions, or those of minors living with them, even though most of the respondents have physical and financial access to a doctor (they are either insured or beneficiaries of a state programme). The result is similar to those reported for SMA in the Euro-Mediterranean region and developing countries, where the overall median proportions of self-medication were 40.9% and 38.8%, respectively (Nepal and Bhatta, 2018). Respondents indicate that they prefer to ask for advice from neighbours or friends with medical education, or rely on previous treatment experience. Such informal approaches can become habits and further exacerbate the problem. Moreover, it is necessary to strengthen the supervision of the behaviour of medical personnel. Even when antibiotics are prescribed by medical practitioners, only half of them give patients written prescriptions and fewer give advice regarding antibiotic therapy. This practice does not contribute to the development of doctor-patient relationships. The results of the study suggest that the population is more careful about the arbitrary use of antibiotics for treating health conditions in minors than for their own needs, although the data obtained are still concerning. However, it should be noted that the study did not reveal excessive or frequent use of antibiotics, as most respondents used them once a year or less often. The fact that the oral administration is most common method is consistent with the practice of self-medication (easy administration). Unfortunately, pharmacies are the primary sources of the antibiotics, just like in other Asian and European countries (Väänänen et al., 2006; Nepal and Bhatta, 2018), despite the prescription reform implemented in the country (an electronic prescription system has been introduced, and regular surveillance is underway (World Health Organization, 2019)). People often lack a clear idea and accurate information about the negative impact of self-medication with antibiotics. Their decisions are not based on scientific evidence. The lack of knowledge about self-medication is the prime reason for mass scale antibiotic resistance tragedy (Rather et al., 2017). The problem looks even more complicated if we consider that only one-third of the respondents were able to correctly identify the antibiotics used or mistakenly think that antibiotics can treat viral or fungal diseases.

All detected circumstances affect the situation in the country regarding antibiotic consumption.

In addition, a strong correlation between secondary vocational education, higher education (bachelor’s degree) or incomplete higher education and factors F1 and F2, identified as part of the study, were expected. Similar trends were observed in European countries (Grigoryan et al., 2010). The rapid technological advances, especially the Internet and related communication systems, have opened new possibilities for searching for information, although there are apparent differences in opportunities to obtain access to this information among people with different educational backgrounds. Also, the correlation was stronger between women and F1. It shows that females had better knowledge about antibiotic use as compared to males, like some Asian countries (Adeel et al., 2023).

### 4.3 Conclusion

While the sale of antimicrobial drugs over-the-counter is prohibited in the country, there are evident challenges in enforcing the law, similar to other Southern and Eastern European and South Asian countries (Grigoryan et al., 2006; Grigoryan et al., 2010; Nepal and Bhatta, 2018). The findings of our study, indicating that pharmacies are the primary source of antibiotics for self-medication, underscore the need to develop effective methods for implementing electronic prescription (E-prescription) systems to curb the over-the-counter sale of antibiotics without a doctor’s prescription.

The study results, revealing that over 50% of the respondents correctly identified the factors determining antibiotic resistance, provide a basis to assume that implementing appropriate measures to raise awareness among the population of Georgia will yield positive results. We believe that, considering the experiences of the United States, Canada, European countries, and Australia (Grigoryan et al., 2006; Väänänen et al., 2006), it is advisable to conduct large-scale public awareness campaigns that provide detailed instructions and emphasize the potential risks of using antimicrobial drugs without medical guidance. This includes countering misconceptions regarding the necessity of antibiotics during minor illnesses, as our research has also indicated. This viewpoint is shared by researchers in South Asian countries (Nepal and Bhatta, 2018).

Public awareness of antibiotics and antimicrobial resistance will be a key objective of the new national strategy (2024–2028) and action plan (2024–2026) on antimicrobial resistance, aligning with approaches in other European countries (Goossens et al., 2005; Väänänen et al., 2006; Grigoryan et al., 2010; Rather et al., 2017), because unlike most other drugs that only impact individual patients, misused antibiotics pose a global risk by increasing the spread of antimicrobial resistance if utilized for self-medication.

## Data Availability

The raw data supporting the conclusion of this article will be made available by the authors, without undue reservation.
