# Supplementary material for: Self-medication with antibiotics in Georgian population
Source: Front Pharmacol. 2024 Feb 21;15:1254817. doi: 10.3389/fphar.2024.1254817 (PMC10915006; doi:10.3389/fphar.2024.1254817)
Supplement: Supplementary file 1 [file Table1.DOCX]

Appendix 3. Questionnaire

Part I. Socio-demographic data

1.Gender

Male

Female

Other (specify) ------------------

2. Age (years)

18 - 25

26-40

41-60

>60

3. Nationality

Georgian

Armenian

Azerbaijanian

Russian

Other (specify) ------------------

4. Education

Primary

Incomplete secondary

Secondary

Secondary vocational

Incomplete high

High:

Undergraduate

Master

Doctoral

5. Marital status

Single

Married

Couple living together

Divorced/living separately

Widow

6. Do you have children (<18 years) living with you?

Yes

No

7. Labor activity

Employed

Self-employed

Unemployed

Pensioner

Other (specify)----------------

8. Income

< 500 GEL per month

500-1000 GEL per month

1000-3000 GEL per month

>3000 GEL per month

Part II. Personal health information

1. Do you have an insurance?

Yes

No

Not sure

1. Are you a beneficiary of the universal health state program or another state program?

Yes

Yes, but I don't remember (which program)

No

Not sure

1. Do you use the services of a family doctor?

Yes

No

Not sure

1. Which chronic disease do you suffer from?

I don't have any chronic diseases

Hypertension

Diabetes, type 2

Cardiovascular disease

Asthma

Chronic kidney disease

Malignant tumor (any localization)

Other (specify) --------------

Part III. Information about the use of antibiotics

1.When was the last time you took an antibiotic?

For the last month

For the last 6 months

During the last year

More than year ago

I never got it

Do not remember

2.If you took antibiotics, was it prescribed by a doctor? (In case of a positive answer to the question, fill in questions 3-5, in case of a negative answer - questions 7-12)

Yes

No

Other (specify) --------------

3.If you took the antibiotic on the doctor's decision, did you get a prescription (electronic or paper -based)? (In case of a positive answer, fill in the 4th question as well)

Yes

No

Do not remember

4.If you took the antibiotic on the doctor's decision, did you use a prescription when buying the antibiotic at the pharmacy?

Yes (asked for and used)

No (not requested and not used)

Do not remember

5.If you took an antibiotic by a doctor's decision, did you get an counseling and advice regarding the use of an antibiotic?

Yes

No

Do not remember

6.Do you think that antibiotics can only be taken with a doctor's prescription?

Yes

No

I don't know

7. If the antibiotic was not prescribed by the doctor, on whose recommendation did you take it?

of a family member who is not a doctor but has a medical education (nurse, pharmacist)

of a family member who does not have a medical education

of a friend or neighbor who is not a doctor but has a medical education (nurse, pharmacist)

of a friend or neighbor who does not have a medical education

I made the decision myself

8. If the antibiotic was not prescribed by the doctor, where did you get the antibiotic?

In the pharmacy

Internet/online pharmacy

A friend/neighbor gave it to me

We had leftovers from the previous treatment in the family

Do not remember

Other (specify)---------

9. If antibiotics were not prescribed by a doctor, for which disease/pathological condition did you take them?

Diseases of the respiratory system

Diseases of the urogenital system

Diseases of the cardiovascular system

Neurological diseases

Diseases of the gastrointestinal system

Tooth injuries/diseases

Other (specify) ----------------

10.Can you name the antibiotic used?

Yes (specify) ----------------

No

I do not remember

11.The route of administration of antibiotics used in this occasion:

Parenteral

Oral

Do not remember

12. By what criteria do you choose an antibiotic for different diseases?

I read the instructions for the medicine

I ask the medical personnel

I have previous treatment experience

I use the experience of a neighbor/friend regarding treatment

The main thing is that it is an antibiotic, it doesn't matter which one it is

Other (specify) ------------

13.Have you used an antibiotic to treat a minor member of your family by your own decision? (If the answer to this question is not pozitive, go to question 18)

Yes

No

I do not remember

14. When was the last time you gave an antibiotic to a minor living with you (if such a case took place)?

For the last month

For the last 6 months

During the last one year

More than a year ago

There was no such case

I do not remember

15.In this occasion, for which disease/pathological condition did you administer it to your minor family member?

Diseases of the respiratory system

Diseases of the urogenital system

Diseases of the cardiovascular system

Neurological diseases

Diseases of the gastrointestinal system

Tooth injuries/diseases

Other (specify) ----------------

16.Can you name the antibiotic used?

Yes (specify)--------------

No

Do not remember

17.The route of administration of antibiotics used in this occasion:

Parenteral

Oral

Do not remember

1. Have the results of antibiotic treatment met your expectations without a doctor's prescription,? (In your opinion, did the treatment end with recovery?)

Yes

No

I don't know

Do not remember

1. In general, how often do you use antibiotics without a doctor's prescription?

Very often (monthly)

Often (once in 3-6 months)

Rarely (once a year)

Very rarely (once every few years)

Never took it

20 In your opinion, antibiotics are used to treat infectious diseases caused by which microorganisms?

Viruses

Bacteria

Fungi

Parasites

All answers are correct

I don’t know

21.Have you ever heard about the threat called antibiotic resistance?

Yes

No

I don't know

22. If yes, what do you think contributes to the development of antibiotic resistance?

Taking antibiotics without a doctor's prescription

Using the wrong dose of antibiotics

Use of antibiotics for an incorrect duration

Treatment with inappropriately selected antibiotics

I don't know

(Note: You can mark multiple answers at once)

22.Do you think that antibiotics should not be dispensed from the pharmacy without a prescription?

Yes

No

I don't know

Part IV. Additional questionnaire for those who independently use antibiotics to treat themselves or a minor living with them

On a scale of 1 to 5 (1 - strongly disagree, 2 - disagree, 3 - neither agree nor disagree, 4 - mostly agree, 5 - completely agree) indicate how much you agree with the following statements:

a) Usually, I use antibiotics on my own due to lack of time to visit a doctor

I strongly disagree;

I disagree;

I neither agree nor disagree;

I mostly agree

I strongly agree

b) I usually use antibiotics on my own due to their easy availability at pharmacies

I strongly disagree;

I disagree;

I neither agree nor disagree;

I mostly agree

I strongly agree

c) I usually,use antibiotics on my own because of the high cost of seeing a doctor

I strongly disagree;

I disagree;

I neither agree nor disagree;

I mostly agree

I strongly agree

d) I usually self-administer antibiotics for simple signs and symptoms of the illness (i.e. I do not consider it necessary to consult a doctor for this reason)

I strongly disagree;

I disagree;

I neither agree nor disagree;

I mostly agree

I strongly agree

e) I usually use antibiotics on my own, based on previous experience with the same antibiotic

I strongly disagree;

I disagree;

I neither agree nor disagree;

I mostly agree

I strongly agree

f) I usually use antibiotics on my own, due to lack of trust in doctors

I strongly disagree;

I disagree;

I neither agree nor disagree;

I mostly agree

I strongly agree

g) I usually use antibiotics on my own if I ever (or the baby does) have diarrhea, including when traveling or on vacation abroad

I strongly disagree;

I disagree;

I neither agree nor disagree;

I mostly agree

I strongly agree

h) I usually self-administer antibiotics for sore throat/cold/cough right away to prevent further complications

I strongly disagree;

I disagree;

I neither agree nor disagree;

I mostly agree

I strongly agree

t) Usually, I use antibiotics on my own for genitourinary infection.

I strongly disagree;

I disagree;

I neither agree nor disagree;

I mostly agree

I strongly agre

j) Usually, I use antibiotics on my own to prevent diseases

I strongly disagree;

I disagree;

I neither agree nor disagree;

I mostly agree

I strongly agree

k) In case of a positive answer to the previous question, please indicate which disease you use antibiotics for prevention?

------------------------
