# Supplementary material for: Self-medication with antibiotics in Georgian population
Source: Front Pharmacol. 2024 Feb 21;15:1254817. doi: 10.3389/fphar.2024.1254817 (PMC10915006; doi:10.3389/fphar.2024.1254817)
Supplement: Supplementary file 2 [file Table2.DOCX]

Appendix 2

Table 1. Do you have an insurance?

|  | Frequency | Percent |
| --- | --- | --- |
| Yes | 464 | 62.5 |
| No | 278 | 37.5 |
| Total | 742 | 100.0 |

Table 2. Are you a beneficiary of the State Universal Health Care program or another state program?

|  | Frequency | Percent |
| --- | --- | --- |
| Yes | 287 | 38.7 |
| No | 455 | 61.3 |
| Total | 742 | 100.0 |

Table 3. Do you use the services of a family doctor?

|  | Frequency | Percent |
| --- | --- | --- |
| Yes | 542 | 73.1 |
| No | 200 | 26.9 |
| Total | 742 | 100.0 |

Table 4. When was the last time you took an antibiotic?

|  | | Frequency | Percent |
| --- | --- | --- | --- |
| Valid | For the last month | 91 | 12,2 |
|  | For the last 6 months | 175 | 23,6 |
|  | During the last year | 91 | 12,3 |
|  | More than year ago | 247 | 33,3 |
|  | Never took it | 14 | 1,8 |
|  | Do not remember. | 125 | 16,9 |
|  | Total | 742 | 100,0 |

Table 5. If you took antibiotics, was it prescribed by a doctor? (In case of a positive answer to the question, fill in questions 3-5, in case of a negative answer - questions 7-12)

|  | Frequency | Percent |
| --- | --- | --- |
| Yes | 565 | 76.2 |
| No | 177 | 23.8 |
| Total | 742 | 100.0 |

Table 6. If you took the antibiotic on the doctor's decision, did you get a prescription (electronic or paper based)? (In case of a positive answer, fill in the 4th question as well)

|  | | Frequency | Percent |
| --- | --- | --- | --- |
| Valid | Yes | 361 | 48,6 |
|  | No | 139 | 18,7 |
|  | Do not remember | 66 | 8,9 |
|  | Total | 565 | 76,2 |
| Missing | System | 177 | 23,8 |
| Total | | 742 | 100,0 |

Table 7. If you took the antibiotic on the doctor's decision, did you use a prescription when buying the antibiotic at the pharmacy?

|  | | Frequency | Percent |
| --- | --- | --- | --- |
| Valid | Yes (asked for and used) | 289 | 39,0 |
|  | No (not requested and not used) | 10 | 1,3 |
|  | Do not remember | 62 | 8,3 |
|  | Total | 361 | 48,6 |
| Missing | System | 381 | 51,4 |
| Total | | 742 | 100,0 |

Table 8. If you took an antibiotic by a doctor's decision, did you get an counseling and advice regarding the use of an antibiotic?

|  | | Frequency | Percent |
| --- | --- | --- | --- |
| Valid | Yes | 291 | 39,2 |
|  | No | 28 | 3,8 |
|  | Do not remember | 42 | 5,6 |
|  | Total | 361 | 48,6 |
| Missing | System | 381 | 51,4 |
| Total | | 742 | 100,0 |

Table 9. Do you think that antibiotics can only be taken with a doctor's prescription?

|  | Frequency | Percent |
| --- | --- | --- |
| Yes | 650 | 87.5 |
| No | 92 | 12.5 |
| Total | 742 | 100.0 |

Table 10.If the antibiotic was not prescribed by the doctor, on whose recommendation did you take it?

|  | | Frequency | Percent | Valid Percent |
| --- | --- | --- | --- | --- |
| Valid | I made the decision myself | 113 | 15,2 | 63,8 |
|  | Of a friend or neighbor who is not a doctor but has a medical education (nurse, pharmacist) | 32 | 4,3 | 17,9 |
|  | Of a friend or neighbor who does not have a medical education | 32 | 4,4 | 18,3 |
|  | Total | 177 | 23,8 | 100,0 |
| Missing | System | 565 | 76,2 |  |
| Total | | 742 | 100,0 |  |

Table 11.If the antibiotic was not prescribed by the doctor, where did you get the antibiotic?

|  | | Frequency | Percent | Valid Percent |
| --- | --- | --- | --- | --- |
| Valid | In the pharmacy | 114 | 15,4 | 64,7 |
|  | Internet/online pharmacy | 5 | ,6 | 2,6 |
|  | We had leftovers from the previous treatment in the family | 18 | 2,5 | 10,4 |
|  | I don't remember when I last took an antibiotic | 5 | ,6 | 2,6 |
|  | Do not remember | 35 | 4,7 | 19,7 |
|  | Total | 177 | 23,8 | 100,0 |
| Missing | System | 565 | 76,2 |  |
| Total | | 742 | 100,0 |  |

Table 12.If antibiotics were not prescribed by a doctor, for which disease/pathological condition did you take them?

|  | | Frequency | Percent | Valid Percent |
| --- | --- | --- | --- | --- |
| Valid | Diseases of the respiratory system | 69 | 9,4 | 39,3 |
|  | Diseases of the urogenital system | 7 | ,9 | 3,7 |
|  | Diseases of the cardiovascular system | 1 | ,2 | ,8 |
|  | Headache | 4 | ,5 | 2,1 |
|  | Neurological diseases | 1 | ,1 | ,5 |
|  | Diseases of the gastrointestinal system | 19 | 2,6 | 10,9 |
|  | Tooth injuries/diseases | 29 | 3,9 | 16,3 |
|  | Other | 3 | ,4 | 1,7 |
|  | Do not remember | 44 | 5,9 | 24,7 |
|  | Total | 177 | 23,8 | 100,0 |
| Missing | System | 565 | 76,2 |  |
| Total | | 742 | 100,0 |  |

Table 13. The route of administration of antibiotics used

|  | | Frequency | Percent |
| --- | --- | --- | --- |
| Valid | Oral | 143 | 80,8 |
|  | Parenteral | 3 | 1,7 |
|  | Do not remember | 31 | 17,5 |
|  | Total | 177 | 100,0 |

Table 14. By what criteria do you choose an antibiotic for different diseases?

|  | | Frequency | Percent |
| --- | --- | --- | --- |
| Valid | I read the instructions for the medicine | 51 | 28,8 |
|  | I ask the medical personnel | 45 | 25,4 |
|  | I have previous treatment experience | 58 | 32,8 |
|  | I use the experience of a neighbor/friend regarding treatment | 5 | 2,8 |
|  | The main thing is that it is an antibiotic, it doesn't matter which one it is | 2 | 1,1 |
|  | Other („ I use it with the doctor’s prescription“) | 9 | 5,1 |
|  | Other („Do not use at all“) | 7 | 4,0 |
|  | Total | 177 | 100,0 |

Table 15. Have you used an antibiotic to treat a minor member of your family by your own decision? (If the answer to this question is not pozitive, go to question 18)

|  | | Frequency | Percent |
| --- | --- | --- | --- |
| Valid | Yes | 94 | 12,7 |
|  | No | 627 | 84,4 |
|  | Do not remember | 21 | 2,9 |
|  | Total | 742 | 100,0 |

Table 16. When was the last time you gave an antibiotic to a minor living with you (if such a case took place)?

|  | | Frequency | Percent | Valid Percent |
| --- | --- | --- | --- | --- |
| Valid | For the last month | 9 | 1,2 | 9,1 |
|  | For the last 6 months | 31 | 4,1 | 32,5 |
|  | During the last one year | 12 | 1,6 | 12,4 |
|  | More than a year ago | 28 | 3,7 | 29,6 |
|  | Do not remember | 15 | 2,1 | 16,5 |
|  | Total | 94 | 12,7 | 100,0 |
| Missing | System | 648 | 87,3 |  |
| Total | | 742 | 100,0 |  |

Table 17. In this occasion, for which disease/pathological condition did you administer it to your minor family member?

|  | | Frequency | Percent | Valid Percent |
| --- | --- | --- | --- | --- |
| Valid | Diseases of the respiratory system | 69 | 9,2 | 73,0 |
|  | Diseases of the urogenital system | 9 | 1,2 | 9,2 |
|  | Diseases of the cardiovascular system |  | ,1 | ,5 |
|  | Diseases of the gastrointestinal system | 5 | ,7 | 5,2 |
|  | Tooth injuries/diseases | 4 | ,5 | 4,1 |
|  | Do not remember | 8 | 1,0 | 8,0 |
|  | Total | 94 | 12,7 | 100,0 |
| Missing | System | 648 | 87,3 |  |
| Total | | 742 | 100,0 |  |

Table 18. The route of administration of antibiotics used for minors

|  | | Frequency | Percent | Valid Percent |
| --- | --- | --- | --- | --- |
| Valid | Parenteral | 4 | ,6 | 4,8 |
|  | Oral | 86 | 11,6 | 91,2 |
|  | Do not remember | 4 | ,5 | 4,0 |
|  | Total | 94 | 12,7 | 100,0 |
| Missing | System | 648 | 87,3 |  |
| Total | | 742 | 100,0 |  |

Table 19.Use of antibiotics without a doctor's prescription in total (including for the treatment of a minor)

|  | Frequency | Percent |
| --- | --- | --- |
| No | 500 | 67.4 |
| Yes | 242 | 32.6 |
| Total | 742 | 100.0 |

Table 20. Have the results of antibiotic treatment met your expectations without a doctor's prescription,? (In your opinion, did the treatment end with recovery?)

|  | | Frequency | Percent | Valid Percent |
| --- | --- | --- | --- | --- |
| Valid | Yes | 97 | 13,1 | 40,2 |
|  | No | 3 | ,4 | 1,1 |
|  | Do not remember | 142 | 19,2 | 58,7 |
|  | Total | 242 | 32,6 | 100,0 |
| Missing | System | 500 | 67,4 |  |
| Total | | 742 | 100,0 |  |

Table 21. In general, how often do you use antibiotics without a doctor's prescription?

|  | | Frequency | Percent | Valid Percent |
| --- | --- | --- | --- | --- |
| Valid | Very often (monthly) | 7 | ,9 | 2,8 |
|  | Often (once in 3-6 months) | 9 | 1,2 | 3,5 |
|  | Rarely (once a year) | 57 | 7,7 | 23,6 |
|  | Very rarely (once every few years) | 130 | 17,6 | 53,9 |
|  | Never took it | 39 | 5,3 | 16,1 |
|  | Total | 242 | 32,6 | 100,0 |
| Missing | System | 500 | 67,4 |  |
| Total | | 742 | 100,0 |  |

Table 22. In your opinion, antibiotics are used to treat infectious diseases caused by which microorganisms?

|  | | Frequency | Percent |
| --- | --- | --- | --- |
| Valid | Viruses | 121 | 16,3 |
|  | Bacteria | 381 | 51,4 |
|  | Fungi | 10 | 1,3 |
|  | Parasites | 7 | ,9 |
|  | All answers are correct | 150 | 20,3 |
|  | Do not know | 73 | 9,9 |
|  | Total | 742 | 100,0 |

Table 23. If yes, what do you think contributes to the development of antibiotic resistance?

|  |  | |
| --- | --- | --- |
|  | Count | Column N % |
| Taking antibiotics without a doctor's prescription | 407 | 54,8% |
| Using the wrong dose of antibiotics | 411 | 55,4% |
| Use of antibiotics for an incorrect duration | 371 | 49,9% |
| Treatment with inappropriately selected antibiotics | 468 | 63,1% |
| Do not know | 88 | 11,8% |

Table 24. (Q1). Usually, I use antibiotics on my own due to lack of time to visit a doctor

|  | | Frequency | Percent | Valid Percent |
| --- | --- | --- | --- | --- |
| Valid | Strongly disagree | 45 | 6,1 | 18,6 |
|  | Disagree | 77 | 10,4 | 31,9 |
|  | Neither agree nor disagree | 71 | 9,5 | 29,1 |
|  | Mostly agree | 43 | 5,8 | 17,6 |
|  | Strongly agree | 6 | ,9 | 2,7 |
|  | Total | 242 | 32,6 | 100,0 |
| Missing | System | 500 | 67,4 |  |
| Total | | 742 | 100,0 |  |

Table 25.(Q 2). I usually use antibiotics on my own due to their easy availability at pharmacies

|  | | Frequency | Percent | Valid Percent |
| --- | --- | --- | --- | --- |
| Valid | Strongly disagree | 36 | 4,9 | 14,9 |
|  | Disagree | 76 | 10,2 | 31,2 |
|  | Neither agree nor disagree | 76 | 10,2 | 31,4 |
|  | Mostly agree | 48 | 6,5 | 19,9 |
|  | Strongly agree | 6 | ,9 | 2,6 |
|  | Total | 242 | 32,6 | 100,0 |
| Missing | System | 500 | 67,4 |  |
| Total | | 742 | 100,0 |  |

Table 26.(Q3). I usually,use antibiotics on my own because of the high cost of seeing a doctor

|  | | Frequency | Percent | Valid Percent |
| --- | --- | --- | --- | --- |
| Valid | Strongly disagree | 37 | 5,0 | 15,3 |
|  | Disagree | 68 | 9,2 | 28,1 |
|  | Neither agree nor disagree | 70 | 9,5 | 29,0 |
|  | Mostly agree | 48 | 6,4 | 19,6 |
|  | Strongly agree | 19 | 2,6 | 8,0 |
|  | Total | 242 | 32,6 | 100,0 |
| Missing | System | 500 | 67,4 |  |
| Total | | 742 | 100,0 |  |

Table 27. (Q4). I usually self-administer antibiotics for simple signs and symptoms of the illness (i.e. I do not consider it necessary to consult a doctor for this reason)

|  | | Frequency | Percent | Valid Percent |
| --- | --- | --- | --- | --- |
| Valid | Strongly disagree | 40 | 5,4 | 16,5 |
|  | Disagree | 65 | 8,7 | 26,6 |
|  | Neither agree nor disagree | 62 | 8,3 | 25,5 |
|  | Mostly agree | 65 | 8,8 | 26,9 |
|  | Strongly agree | 11 | 1,4 | 4,4 |
|  | Total | 242 | 32,6 | 100,0 |
| Missing | System | 500 | 67,4 |  |
| Total | | 742 | 100,0 |  |

Table 28.(Q5). I usually use antibiotics on my own, based on previous experience with the same antibiotic

|  | | Frequency | Percent | Valid Percent |
| --- | --- | --- | --- | --- |
| Valid | Strongly disagree | 21 | 2,9 | 8,8 |
|  | Disagree | 49 | 6,5 | 20,0 |
|  | Neither agree nor disagree | 46 | 6,2 | 19,0 |
|  | Mostly agree | 112 | 15,1 | 46,2 |
|  | Strongly agree | 15 | 2,0 | 6,0 |
|  | Total | 242 | 32,6 | 100,0 |
| Missing | System | 500 | 67,4 |  |
| Total | | 742 | 100,0 |  |

Table 29. (Q6). I usually use antibiotics on my own, due to lack of trust in doctors

|  | | Frequency | Percent | Valid Percent |
| --- | --- | --- | --- | --- |
| Valid | Strongly disagree | 53 | 7,2 | 21,9 |
|  | Disagree | 104 | 14,0 | 42,9 |
|  | Neither agree nor disagree | 55 | 7,4 | 22,7 |
|  | Mostly agree | 25 | 3,4 | 10,5 |
|  | Strongly agree | 5 | ,7 | 2,0 |
|  | Total | 242 | 32,6 | 100,0 |
| Missing | System | 500 | 67,4 |  |
| Total | | 742 | 100,0 |  |

Table 30. (Q7). I usually use antibiotics on my own if I ever (or the baby does) have diarrhea, including when traveling or on vacation abroad

|  | | Frequency | Percent | Valid Percent |
| --- | --- | --- | --- | --- |
| Valid | Strongly disagree | 31 | 4,2 | 12,9 |
|  | Disagree | 90 | 12,1 | 37,0 |
|  | Neither agree nor disagree | 67 | 9,1 | 27,7 |
|  | Mostly agree | 47 | 6,3 | 19,4 |
|  | Strongly agree | 7 | 1,0 | 2,9 |
|  | Total | 242 | 32,6 | 100,0 |
| Missing | System | 500 | 67,4 |  |
| Total | | 742 | 100,0 |  |

Table 31. (Q8). I usually self-administer antibiotics for sore throat/cold/cough right away to prevent further complications

|  | | Frequency | Percent | Valid Percent |
| --- | --- | --- | --- | --- |
| Valid | Strongly disagree | 33 | 4,5 | 13,7 |
|  | Disagree | 75 | 10,1 | 31,1 |
|  | Neither agree nor disagree | 50 | 6,7 | 20,5 |
|  | Mostly agree | 73 | 9,9 | 30,3 |
|  | Strongly agree | 11 | 1,5 | 4,5 |
|  | Total | 242 | 32,6 | 100,0 |
| Missing | System | 500 | 67,4 |  |
| Total | | 742 | 100,0 |  |

Table 32. (Q9.) Usually, I use antibiotics on my own for genitourinary infection

|  | | Frequency | Percent | Valid Percent |
| --- | --- | --- | --- | --- |
| Valid | Strongly disagree | 49 | 6,6 | 20,3 |
|  | Disagree | 92 | 12,4 | 38,0 |
|  | Neither agree nor disagree | 56 | 7,5 | 23,1 |
|  | Mostly agree | 39 | 5,3 | 16,3 |
|  | Strongly agree | 5 | ,7 | 2,3 |
|  | Total | 242 | 32,6 | 100,0 |
| Missing | System | 500 | 67,4 |  |
| Total | | 742 | 100,0 |  |

Table 33. (Q10). Usually, I use antibiotics on my own to prevent diseases

|  | | Frequency | Percent | Valid Percent |
| --- | --- | --- | --- | --- |
| Valid | Strongly disagree | 84 | 11,4 | 34,8 |
|  | Disagree | 90 | 12,1 | 37,2 |
|  | Neither agree nor disagree | 41 | 5,5 | 16,8 |
|  | Mostly agree | 18 | 2,5 | 7,6 |
|  | Strongly agree | 9 | 1,2 | 3,7 |
|  | Total | 242 | 32,6 | 100,0 |
| Missing | System | 500 | 67,4 |  |
| Total | | 742 | 100,0 |  |
