# Supplementary material for: Self-medication with antibiotics in Georgian population
Source: Front Pharmacol. 2024 Feb 21;15:1254817. doi: 10.3389/fphar.2024.1254817 (PMC10915006; doi:10.3389/fphar.2024.1254817)
Supplement: Supplementary file 3 [file Table3.docx]

**Appendix 1**

**Tables**

Table1.Gender

| Gender | Frequency | Percent |
| --- | --- | --- |
| Female | 396 | 53,4 |
| Male | 346 | 46,6 |
| Total | 742 | 100,0 |

Table 2. Age (years)

| Age (years) | Frequency | Percent |
| --- | --- | --- |
| 18 - 24 | 139 | 18,7 |
| 25- 40 | 189 | 25,5 |
| 41 - 60 | 239 | 32,2 |
| > 60 | 175 | 23,6 |
| Total | 742 | 100,0 |
|  |  |  |

Table 3. Nationality

| Nationality | Frequency | Percent |
| --- | --- | --- |
| Georgian | 730 | 98,4 |
| Other | 12 | 1,6 |
| Total | 742 | 100,0 |

Table 4. Marital status

| Marital status | Frequency | Percent |
| --- | --- | --- |
| Married | 460 | 62,0 |
| living separately | 282 | 38,0 |
| Total | 742 | 100,0 |

Table 5. Children (<18 years) living with respondents

| Do you have children (<18 years) living with you? | Frequency | Percent |
| --- | --- | --- |
| Yes | 335 | 45,2 |
| No | 407 | 54,8 |
| Total | 742 | 100,0 |

Table 6. Labor activity

| Labor activity | Frequency | Percent |
| --- | --- | --- |
| Employed | 579 | 78,0 |
| Unemployed | 163 | 22,0 |
| Total | 742 | 100,0 |

Table 7. Income

| Income | Frequency | Percent |
| --- | --- | --- |
| < 500 GEL per month | 134 | 18,1 |
| 500-1000 GEL per month | 241 | 32,5 |
| 1000-3000 GEL per month | 226 | 30,4 |
| >3000 GEL per month | 141 | 19,0 |
| Total | 742 | 100,0 |

Table 8. A factor analysis on the variables of part 4 of the questionnaire.

| N | The variables of part 4 of the questionnaire | Component | |
| --- | --- | --- | --- |
|  |  | F1 | F2 |
| 1.  2.  3.  4.  5.  6.  7.  8.  9.  10. | Usually, I use antibiotics on my own, due to the lack of time to visit a doctor.  I usually use antibiotics on my own due to their easy (over the counter) availability at pharmacies  I usually use antibiotics on my own because of the high cost of seeing a doctor  I usually use antibiotics for a simple signs and symptoms of illness (i.e., I don't see the need to consult a doctor for this reason).  I usually use antibiotics on my own, based on my previous experience with the same antibiotics  I usually use antibiotics on my own due to lack of trust in doctors  I usually use antibiotics on my own if I ever (or the baby does) have diarrhea, including when traveling abroad or on vacation  I usually self-administer antibiotics for sore throats/colds/coughs right away to prevent further complications  Usually, I use antibiotics on my own for genitourinary infection  Usually, I use antibiotics on my own to prevent diseases | 78.6%  80.4%  73.5%  76.4%  72.9%  33.2%  54.3%  44.4%  7.3%  13.8% | 8.7%  12.5%  28.1%  31.9%  32.7%  61.9%  50.1%  66.7%  73.3%  79.4% |

Table 9. A correlation between factor F1 ("personal experience") &F2 ("lack of trust in medical practitioners" ) and gender (ANOVA Table)

|  | | Sum of Squares | df | Mean Square | F | Sig. |
| --- | --- | --- | --- | --- | --- | --- |
| FAC1_1 REGR factor score 1 for analysis 1 * D1 1. Gender | Between (Combined) Groups | 3.762 | 1 | 3.762 | 3.805 | .042 |
| FAC2_1 REGR factor score 2 for analysis 1 * D1 1. Gender | Between (Combined) Groups | 1.018 | 1 | 1.018 | 1.018 | 0.314 |

Table 10.

| Gender | FAC1_1 REGR factor score 1 for analysis 1 | FAC2_1 REGR factor score 2 for analysis 1 |
| --- | --- | --- |
| Female | -.1194319 | -.0621419 |
| Male | .1300136 | .0676476 |
| Total | .0000000 | .0000000 |

Table 11. A correlation between factor F1 ("personal experience" ) &F2 ("lack of trust in medical practitioners") and age (ANOVA Table)

|  |  | Sum of Squares | df | Mean Square | F | Sig. |
| --- | --- | --- | --- | --- | --- | --- |
| FAC1_1 REGR factor score 1 for analysis 1 * D2 2. Age (years) | Between (Combined) Groups | .158 | 3 | .053 | .052 | .984 |
| FAC2_1 REGR factor score 2 for analysis 1 * D2 2. Age (years) | Between (Combined) Groups | 7.907 | 3 | 2.636 | 2.691 | .047 |

Table 12.

| Gender (years) | FAC1_1 REGR factor score 1 for analysis 1 | FAC2_1 REGR factor score 2 for analysis 1 |
| --- | --- | --- |
| 18-24 | .0551074 | .3052518 |
| 25-40 | .0094870 | -.1117226 |
| 41-60 | -.0108169 | -.1568369 |
| >60 | -.0259672 | .1773421 |
| Total | .0000000 | .0000000 |

Table 13. A correlation between factor F1 ("personal experience" ) &F2 ("lack of trust in medical practitioners") and education (ANOVA Table).

|  | | Squares | df | Square | F | Sig. |
| --- | --- | --- | --- | --- | --- | --- |
| FAC1_1 REGR factor score 1 for analysis 1 * D4 4. education | Between (Combined) Groups | 10.782 | 5 | 2.156 | 2.210 | .044 |
| FAC2_1 REGR factor score 2 for analysis 1 * D4 4. education | Between (Combined) Groups | 34.583 | 5 | 6.917 | 7.907 | .000 |

Table 14.

| Education | FAC1_1 REGR factor score 1 for analysis 1 | FAC2_1 REGR factor score 2 for analysis 1 |
| --- | --- | --- |
| Secondary education | -.17 | -.45 |
| Secondary professional | .38 | .65 |
| Incomplete higher | -.64 | 1.09 |
| Bachelor | .12 | -.09 |
| Master | .02 | -.07 |
| Ph.D. | -.17 | -.58 |
| Total | .00 | .00 |
